# Tryptophan catabolism in *Pseudomonas aeruginosa* and potential for inter-kingdom relationship

**DOI:** 10.1186/s12866-016-0756-x

**Published:** 2016-07-08

**Authors:** Perrine Bortolotti, Benjamin Hennart, Camille Thieffry, Guillaume Jausions, Emmanuel Faure, Teddy Grandjean, Marion Thepaut, Rodrigue Dessein, Delphine Allorge, Benoit P. Guery, Karine Faure, Eric Kipnis, Bertrand Toussaint, Audrey Le Gouellec

**Affiliations:** Université Lille CHU Lille, EA 7366 - Recherche translationnelle: relations hôte pathogènes, F-59000 Lille, France; Laboratoire de Toxicologie - Pôle de Biologie-Pathologie-Génétique - CHRU de Lille - France, EA4483 - IMPECS, Université Lille Nord de France, Lille, France; Translational host pathogen research group, Faculté de Médecine de Lille UDSL, Univ Lille Nord de France, Lille, France; Faculté de Médecine de Lille UDSL, Univ Lille Nord de France, Lille, France; Laboratoire TIMC-TheREx (UMR5525 CNRS-UGA) Université Grenoble Alpes, Faculté de médecine, La Tronche, France; Unité médicale de Biochimie des enzymes et des protéines, CHUGA de Grenoble , CS10207, Grenoble, 38043 Rhone alpes France

**Keywords:** *Pseudomonas aeruginosa*, Kynurenine, Kynurenic acid, Tryptophan, Anthranilate

## Abstract

**Background:**

*Pseudomonas aeruginosa (Pa)* is a Gram-negative bacteria frequently involved in healthcare-associated pneumonia with poor clinical outcome. To face the announced post-antibiotic era due to increasing resistance and lack of new antibiotics, new treatment strategies have to be developed. Immunomodulation of the host response involved in outcome could be an alternative therapeutic target in *Pa*-induced lung infection. Kynurenines are metabolites resulting from tryptophan catabolism and are known for their immunomodulatory properties. *Pa* catabolizes tryptophan through the kynurenine pathway. Interestingly, many host cells also possess the kynurenine pathway, whose metabolites are known to control immune system homeostasis. Thus, bacterial metabolites may interfere with the host’s immune response. However, the kynurenine pathway in *Pa*, including functional enzymes, types and amounts of secreted metabolites remains poorly known. Using liquid chromatography coupled to mass spectrometry and different strains of *Pa*, we determined types and levels of metabolites produced by *Pa* ex vivo in growth medium, and the relevance of this production in vivo in a murine model of acute lung injury.

**Results:**

Ex vivo, *Pa* secretes clinically relevant kynurenine levels (μM to mM). *Pa* also secretes kynurenic acid and 3-OH-kynurenine, suggesting that the bacteria possess both a functional kynurenine aminotransferase and kynurenine monooxygenase. The bacterial kynurenine pathway is the major pathway leading to anthranilate production both ex vivo and in vivo. In the absence of the anthranilate pathway, the kynurenine pathway leads to kynurenic acid production.

**Conclusion:**

*Pa* produces and secretes several metabolites of the kynurenine pathway. Here, we demonstrate the existence of new metabolic pathways leading to synthesis of bioactive molecules, kynurenic acid and 3-OH-kynurenine in *Pa*. The kynurenine pathway in *Pa* is critical to produce anthranilate, a crucial precursor of some *Pa* virulence factors*.* Metabolites (anthranilate, kynurenine, kynurenic acid) are produced at sustained levels both ex vivo and in vivo leading to a possible immunomodulatory interplay between bacteria and host. These data may imply that pulmonary infection with bacteria highly expressing the kynurenine pathway enzymes could influence the equilibrium of the host’s tryptophan metabolic pathway, known to be involved in the immune response to infection. Further studies are needed to explore the effects of these metabolic changes on the pathophysiology of *Pa* infection.

**Electronic supplementary material:**

The online version of this article (doi:10.1186/s12866-016-0756-x) contains supplementary material, which is available to authorized users.

## Background

**Fig. 1:**
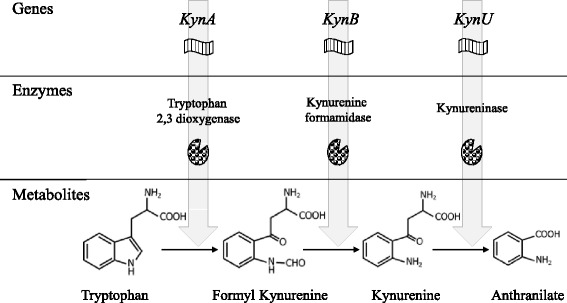
The kynurenine pathway in *Pa.* This figure shows the metabolites resulting from tryptophan catabolism through the kynurenine pathway in *P. aeruginosa*, the three enzymes involved in this metabolic pathway and their related genes

*Pseudomonas aeruginosa (Pa)* is a Gram-negative bacterium frequently involved in healthcare-associated pneumonia and as an opportunistic pathogen in immunocompromised patients [1]. Given its increasing acquired resistance to antibiotics and capacity to adapt to host defenses, *Pa-*induced infections are associated with poor clinical outcome [2]. A better understanding of the metabolic pathways supporting virulence may lead to development of innovative therapeutic strategies [1, 3]. *Pa* belongs to the limited group of pathogens able to metabolize tryptophan into anthranilate through the oxidative kynurenine pathway [2, 4]. Because kynurenine and derivatives are also established bioactive metabolites in humans [5], their bacterial secretion in the microenvironment could play a role in the bacteria-host relationship. Furthermore, anthranilate is a crucial precursor for the Pseudomonas Quinolone Signal (PQS), a signaling molecule of the quorum-sensing system regulating the expression of several virulence factors [6, 7]. Thus, establishing the complete pathways of prokaryotic tryptophan metabolites production during bacterial growth appears necessary from a physiopathological standpoint. Bacteria from the Pseudomonas genus degrade kynurenine either through the kynurenine pathway leading to anthranilate or through the quinolinine pathway, leading to kynurenic acid [8]. In addition, some strains of *Pseudomonas fluorescens* possess a kynurenine monooxygenase (KMO) responsible for 3-OH-kynurenine production [8, 9]. To date, the three known enzymes composing the kynurenine pathway in *Pa* are tryptophan-2,3-dioxygenase (encoded by *kynA)*, kynurenine formamidase (encoded by *kynB*), and kynureninase (encoded by *kynU*), leading respectively to formylkynurenine, kynurenine and anthranilate production (Fig. 1) [4, 10]. However, the concentration range and kinetics of kynurenine production during bacterial growth are poorly known. In addition, whether *Pa* is able to produce kynurenic acid and 3-OH-kynurenine remains unclear. It is currently thought that the *Pa* kynurenine pathway only leads to anthranilate [11]. Anthranilate either results from the conversion of chorismate by anthranilate synthases, or from the degradation of tryptophan via the kynurenine pathway, which is considered to be the predominant pathway for anthranilate synthesis in rich growth medium [12, 13]. However, quantitative assessment of anthranilate production through this pathway has never been performed in *Pa*. Furthermore, whether the kynurenine pathway remains the major source of anthranilate in vivo during infection has never been studied to date. The following experiments aim to determine tryptophan metabolites and their production levels ex vivo during *Pa* growth in rich medium using wild type strain CHA, and *ΔkynA* or *ΔkynU* deletion mutants. We also assessed kynurenine pathway metabolites production in vivo in a murine model of acute lung injury.

## Results

As a preliminary experiment, we compared bacterial growth ex vivo in non-renewed LB medium between wild type and mutant strains, to assess the fitness of mutant strains. Bacterial growth was identical for each strain, strongly suggesting that bacterial fitness is similar (Table 1).

**Table 1:** Bacterial growth assessment of CHA strain and mutants

	CHA	CHA∆kynU	CHA∆kynA
Qx expo (h^−1^)	1.29	1.22	1.34
Generation time (min)	32	34	31

Qx expo refers to the growth rate during exponential growth (h^−1^). Generation time refers to the doubling time of bacterial population during the exponential phase of growth (minutes). One experiment representative of three

### The kynurenine pathway in *Pa* produces high-level anthranilate ex vivo

**Fig. 2:**
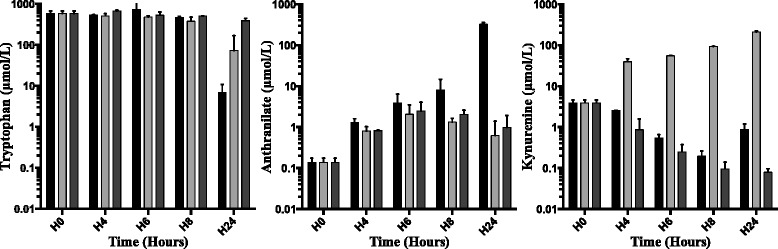
Tryptophan, anthranilate, and L-kynurenine concentrations ex vivo*.* Tryptophan, anthranilate, and L-kynurenine concentrations in growth medium supernatants of CHA strain (black), CHA∆kynA strain (dark gray) and CHA∆kynU strain (light gray) as determined by UPLC-MS-MS. All data from two experiments in duplicates. Error bars represent means +/− SD

To explore the ability of *Pa* to produce anthranilate, we assessed concentrations of tryptophan, kynurenine and anthranilate during bacterial growth (i.e. H4, H6, H8 and H24) in non-renewed LB growth medium of *Pa* strains using an UPLC-MS/MS system. To investigate the role of the kynurenine pathway in anthranilate production, we assessed metabolite concentrations in growth medium of CHA, compared with *kynA* gene deleted strain lacking tryptophan-2,3-dioxygenase (CHA∆kynA) theoretically unable to produce kynurenines, and a *kynU* gene deleted strain (CHA∆kynU), lacking kynureninase, unable to degrade kynurenines and theoretically overproducing them via the KynR-positive transcriptional loop [11]. We show that *Pa* (strain CHA) is able to produce anthranilate ex vivo from 4 h of culture and anthranilate accumulates until 100 μM concentration after 24 h of culture for a bacterial count of 2.10^9^ CFU/ml (Fig. 2). This production is impaired when the *kynA* or *kynU* genes are deleted, leading to a hundred-fold decrease in metabolite concentrations at 24 h. During the first 8 h, tryptophan concentration is stable then decreases in supernatant for CHA strain, but in a lesser extent in CHA∆kynU and not in CHA∆kynA. The stability of tryptophan concentration levels over the first eight hours is consistent with this ability of *Pa* to synthetize tryptophan through its tryptophan synthase, encoded by *trpA/B genes*. The stability of tryptophan concentrations over 24 h in CHA∆kynA growth medium compared with the hundred-fold decrease observed with the wild type strain confirm the essential role of the kynurenine pathway in tryptophan catabolism, given that both strains have the same ability to synthesize tryptophan.

### *Pa* is able to produce kynurenic acid

**Fig. 3:**
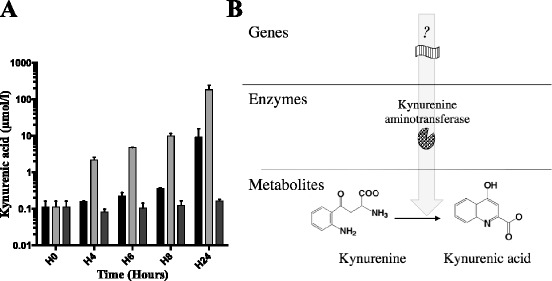
Kynurenic acid concentrations ex vivo and hypothetical metabolic pathway. **a** Kynurenic acid concentrations in CHA (black), CHA∆kynA (dark gray) and CHA∆kynU (light gray) strains growth medium supernatants. All data from two experiments in duplicates. Error bars represent means +/− SD. **b** Hypothetical metabolic pathway responsible for bacterial synthesis of kynurenic acid

We aimed to determine whether *Pa* was able to produce kynurenic acid during growth in rich medium. Using the same protocol, we assessed kynurenic acid concentrations in culture supernatants of CHA, CHA∆kynA and CHA∆kynU strains at H4, H6, H8 and H24. CHA produces kynurenic acid at μM levels, at inoculum identical to those above (Fig. 3). Conversely, CHA∆kynA is unable to produce kynurenic acid, whereas CHA∆kynU overproduces it, reaching 100 μM at H24. This overproduction of kynurenic acid by CHA∆kynU, taken together with the ten-fold decrease in tryptophan concentration in CHA∆kynU growth medium at 24 h compared to initial tryptophan concentration, suggests that an additional enzymatic pathway is involved in tryptophan degradation when kynureninase is lacking.

These results strongly suggest that *Pa* possesses a functional kynurenine aminotransferase (KAT), leading to kynurenic acid production. In addition, the kynurenic acid pathway seems to be the major pathway for tryptophan catabolism when kynureninase function is impaired. Last, these results prove specifically in *Pa*, that both the anthranilate and the kynurenic acid pathways coexist.

### *Pa* is able to produce 3-OH-kynurenine

**Fig. 4:**
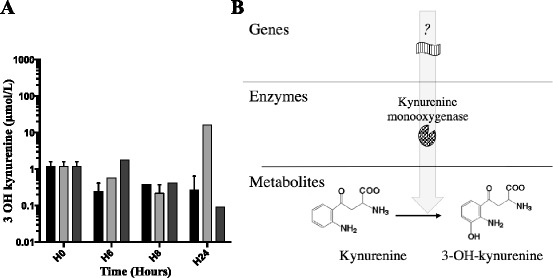
3-OH-kynurenine concentrations ex vivo and hypothetical metabolic pathway. **a** 3-OH-kynurenine concentrations in CHA (black), CHA∆kynA (dark gray) and CHA∆kynU (light gray) strains growth medium supernatants. Representative data from two experiments in duplicates. Error bars represent means +/− SD. **b** Hypothetical metabolic pathway responsible for bacterial synthesis of 3-OH-kynurenine

We aimed to determine whether *Pa* was able to produce 3-OH-kynurenine. We quantified 3-OH-kynurenine in supernatants of growth culture of CHA, CHA∆kynA and CHA∆kynU strains at H6, H8 and H24 (Fig. 4). Concentrations of 3-OH-kynurenine in CHA∆kynU growth medium reached 10 μM at H24. As CHA∆kynU cannot catabolize kynurenine and the production of 3-OH-kynurenine increases in that mutant, these results strongly suggest that *Pa* possesses a functional KMO.

### The kynurenine pathway is critical for anthranilate production in vivo

**Fig 5:**
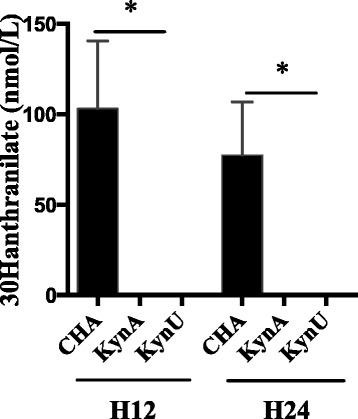
3-OH-anthranilate concentrations in vivo*.* 3-OH-anthranilate concentrations in BAL of infected mice in a 12-hour and a 24-hour infection models with CHA (black), CHA∆kynA and CHA∆kynU strains. All experiments, *n* = 5 mice per group, in duplicates. *p < 0.05. Error bars represent means +/− SEM. Symbols correspond to each sample. Dotted line represents the detection threshold

In an ex vivo model of non-renewed growth culture medium, we show that the kynurenine pathway leads to anthranilate synthesis at high concentrations, and to kynurenic acid production to a lesser extent. In vivo during pneumonia, both the infected host and the bacteria can produce anthranilate and its metabolites through the kynurenine pathway. In this study, we sought to determine the role of the bacterial kynurenine pathway in anthranilate synthesis in vivo in a murine model of acute lung injury. After a 12-hour or a 24-hour acute lung infection induced with CHA, CHA∆kynA or CHA∆kynU strain, mice were sacrificed and bronchoalveolar lavages were performed. Using UPLC-MS/MS, we assessed anthranilate and 3-OH-anthranilate concentrations in BAL (Fig. 5). Anthranilate concentrations were under the detection threshold in the three groups. However, 3-OH-anthranilate was detected in BAL from mice infected with the CHA strain, whereas 3-OH-anthranilate was undetectable in BAL supernatants from mice infected with either CHA∆kynA or CHA∆kynU strains, with detection and quantification limits of respectively 0,5 and 10 nmol/L. As 3-OH-anthranilate directly comes from anthranilate metabolism, this result strongly supports the critical involvement of the bacterial kynurenine pathway in anthranilate production during *Pa*-induced acute lung infection.

### The kynurenine pathway leads to kynurenic acid production in vivo

**Fig. 6:**
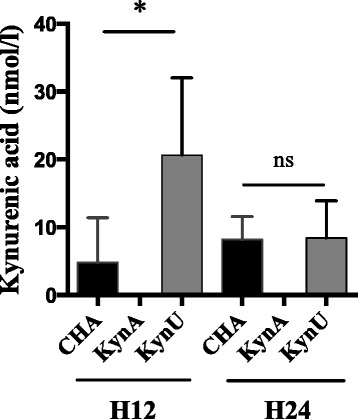
Kynurenic acid concentrations in vivo*.* Kynurenic acid concentrations in BAL of infected mice in 12-hour and 24-hour infection models with CHA (black), CHA∆kynA (dark gray) and CHA∆kynU (light gray) strains. All experiments, *n* = 5 mice per group, in duplicates. *p < 0.05. Error bars represent means +/− SEM

Using the same murine model, we assessed kynurenic acid concentrations in BAL in a 12-hour and 24-hour acute lung infections with CHA, CHA∆kynA or CHA∆kynU strains (Fig. 6). The detection and quantification limits for kynurenic acid were respectively of 0,5 and 10 nmol/L. Kynurenic acid concentrations were significantly increased in BAL of CHA∆kynU infected mice compared with the two other groups at H12. In addition, kynurenic acid concentrations were under the detection threshold in CHA∆kynA induced infection. These results suggest that the bacterial kynurenine pathway leads to significant kynurenic acid production in vivo at H12 post infection.

### Bacterial metabolites of the quinolinine pathway may interfere with the outcome of infection

**Fig. 7:**
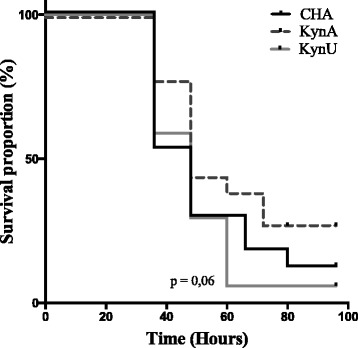
Impact of the bacterial kynurenine pathway on mice survival. Survival of mice infected with lethal inoculum of either CHA (in black), CHA∆kynA (in dash grey), or CHA∆kynU (in light grey) strains. There is a trend toward better survival in mice infected with CHA∆kynA compared with CHA∆kynU strain infection (*p* = 0063). There is no statistically significant difference in survival between mice infected with lethal inoculum of either CHA (in black) and CHA∆kynA (in dash grey), or CHA and CHA∆kynU (in grey) strains. 3 pooled experiments, *n* = 18 per group. Statistic analysis performed with log-rank test

When assessing virulence of each strain through survival of mice during acute lung infection, we showed a trend toward decreased survival in mice infected with CHA∆kynU strain compared to those infected with CHA∆kynA strain (*p* = 0063) (Fig. 7). This result may suggest that kynurenine pathway metabolites could be involved in virulence during acute lung infection. There was no statistically significant difference in survival of mice between strains CHA and CHA∆kynA (*p* = 0,15), and between strains CHA and CHA∆kynU (*p* = 0,57) respectively.

## Discussion

Unlike most bacteria catabolizing tryptophan anaerobically into indole pyruvate and ammonia [14], *Pa* degrades tryptophan through the oxidative kynurenine pathway [15]. The initial step of this pathway is performed by the tryptophan-2,3-dioxygenase, leading to kynurenine synthesis [4]. Previous studies in other organisms or in humans have shown that kynurenine could be catabolized either into kynurenic acid through a KAT, or into anthranilate through a kynureninase [8]. The kynurenine pathway genotype varies with bacterial strains and enzymatic activity depends on growth conditions [8, 16]. In this study, we focused on the potential for cross-talk between host cells and *Pa*. Therefore, we assessed secreted metabolites concentrations in growth medium and not in bacterial lysates. A limitation to our study resides in the possibility that concerning the non-detection of some metabolites one cannot affirm an absence of their production since rapid metabolism or non-secretion could be responsible for low levels.

**Fig. 8:**
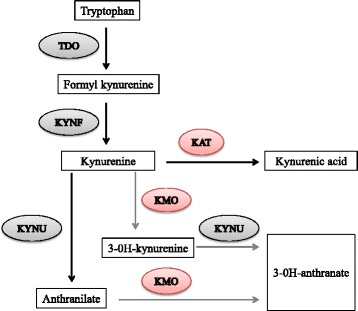
Overview of the proposed enzymatic pathways for tryptophan catabolism in *Pa.* Summary of the proven (dark arrows) and hypothetical (grey arrows) enzymatic reactions composing the kynurenine pathway of *Pa.* Hypothetical enzymes are highlighted in red. TDO: tryptophan-2,3-dioxygenase, KYNF: kynurenine formamidase, KYNU: kynureninase, KAT: kynurenine aminotransferase, KMO: kynurenine monooxygenase

We demonstrate the ability of *Pa* to secrete different tryptophan catabolites from the kynurenine pathway in its environment both ex vivo during growth and in vivo during lung infection (Fig. 8).

Using comparative genomics, Kurnasov et al. reported that *Pa* possesses the genes encoding for the enzymes of the anthranilate pathway, but the authors did not identify genetic support for a quinolinate enzymatic pathway allowing kynurenic acid synthesis [17]. In our study, we show for the first time that *Pa* is able to produce kynurenic acid during growth in rich medium. When *kynU* encoding for kynureninase is lacking, CHA∆kynU produces a large amount of kynurenic acid whereas anthranilate concentration remains stable. Conversely, no kynurenic acid was detected in CHA∆kynA growth medium. A hypothetic pathway involving a reaction between anthranilate and orotic acid to form kynurenic acid has already been invalidated [18]. Taken together, these results suggest that *Pa* expresses a functional KAT, as hypothesized previously for other species of *Pseudomonas* genus bacteria [8, 16]. The identification of KAT homologues in *E. coli,* the hyperthermophilic archaeon *Pyrococcus horikoshii* and human prompted us to search for a homologue in *Pa* [19–21]. Searches using BlastP on Pseudomonas.com with these KAT sequences give several hits. The best match originating from *P. aeruginosa* was from the open reading frame PA3798 (*ybdL*). We also found with lower BLAST scores GntR (PA2320) and PhhC (PA0870), an essential aminotransferase for aromatic amino acid catabolism.

However, the difference between anthranilate and kynurenic acid concentrations suggests that the kynurenine pathway mostly leads to anthranilate production in our ex vivo growth conditions. The absence of sustained increase in anthranilate concentration levels over time after H4 in CHA∆kynA and CHA∆kynU growth media supports this hypothesis. Our results are consistent with previously published data asserting that the kynurenine pathway serves as a critical source of anthranilate in rich medium [13]. The initial ten-fold increase in anthranilate concentration observed for both CHA∆kynU and CHA∆kynA strains suggests the potential involvement of another minor enzymatic pathway for anthranilate synthesis.

The detection of 3-OH-kynurenine in CHA∆kynU growth medium, but not in CHA∆kynA growth medium suggests that *Pa* is able to convert kynurenine into 3-OH-kynurenine, possibly through a KMO. As for the quinolinine enzymatic pathway, to this date no KMO gene has been identified in the *Pa* genome [17]. As mentioned with KAT search, we used KMO sequence from *Pseudomonas fluorescens* strain 17400 (K84HF5) that has 36 % identity and 54 % homology with human KMO, and was experimentally proved as a KMO [9]. We found several hits as PA4190 (PqsL) with 28 % identity on 394 amino acid sequence length and PA3328 a probable FAD-dependent monooxygenase with 26 % identity. This % of identity is not so high considering the species and further experimental researches should be done.

Here we show that *Pa* is able to produce and secrete kynurenine, kynurenic acid and anthranilate at clinically relevant levels (μM to mM) ex vivo in rich medium. We find the same profile of metabolites production using another clinical strain (Additional file 1). Rich medium seems to be the best ex vivo model to simulate the in vivo bacterial growth conditions, as sputum from the lungs of cystic fibrosis patients are rich in amino-acids and form a carbon source to support the growth of *Pa* [22]*.* Further studies are needed in other rich media such as sputum simulation media or clinical sputum samples to better understand the bacterial kynurenine metabolism in vivo.

In vivo, we confirm that the kynurenine pathway is critical for anthranilate production. The increase in 3-OH-anthranilate levels only detected in BAL supernatants from infected mice with CHA strain shows that the bacterial kynurenine pathway is a clinically relevant pathway for anthranilate synthesis in vivo. This result is consistent with the hypothesis of Farrow et al. who speculate that the kynurenine pathway may be particularly important to support PQS production in rich tryptophan environment [13]. In the absence of this anthranilate pathway, infection is associated with kynurenic acid production. The early increase in kynurenic acid in BAL at H12 but not H24 is consistent with data previously published, showing that the *kynA* gene is overexpressed during the first hours of contact between *P. aeruginosa* and immune cells, then returning back to basal transcriptional activity [23].

Interestingly, many eukaryotic cells also produce metabolites from the kynurenine pathway, including anthranilate, 3-OH-anthranilate, kynurenine and kynurenic acid. These molecules possess well-described immunomodulatory functions in mammals [24]. Besides its implication in PQS synthesis, anthranilate, like its metabolite, 3-OH-anthranilate, supports immunomodulatory functions such as dendritic cell activation modulation or T-cell apoptosis [25–28]. Kynurenine and kynurenic acid modulate phagocytic cell functions [23] and recruitment [29], impair dendritic cell immunogenicity [30], and play a role in the polarization of the adaptive immune response [31]. Kynurenine and kynurenic acid also are ligands for the aryl hydrocarbon receptor, a crucial receptor for mucosal immunity [32]. Data generated in vivo may suggest that pulmonary infection with a bacterial strain highly expressing the kynurenine pathway enzymes could impair the equilibrium of the host’s tryptophan metabolism [23]. Thus, the kynurenine pathway could be involved in an inter-kingdom crosstalk between bacteria and their host, and may lead to an imbalance in the immune response to infection [33]. The finding of a trend toward decreased survival in mice infected with CHA∆kynU strain compared to those infected with CHA∆kynA strain, although not statistically significant, may support this hypothesis. However, *P. aeruginosa* possess several redundant virulence factors among which the type three secretion system leading to major cytotoxicity trough secreted endotoxins, particularly in the CHA strain [34]. Considering the metabolites hypothetically involved in bacterial virulence, metabolites produced by the kynurenine pathway are not cytotoxic, even considering the involvement of the PQS, which has not been demonstrated during acute lung infection. Therefore, the role of bacterial kynurenine pathway might rather be an immunomodulatory role, difficult to evidence on outcome alone overwhelmingly due to major bacterial virulence factors [35].

Further research is needed to establish the potential interplay between bacterial and host tryptophan metabolites during infection, possibly paving the way to new immunomodulatory strategies.

## Conclusion

*Pa* is able to produce multiple metabolites from tryptophan at sustained levels both ex vivo and in vivo through catabolism by the kynurenine pathway, including kynurenic acid and 3-OH-kynurenine, which was unknown to this date. The kynurenine pathway in *Pa* is critical to produce anthranilate, a crucial precursor of a *Pseudomonas* virulence factor, the Pseudomonas Quinolone Signal*.* When the anthranilate pathway is impaired, *Pa* tryptophan metabolism produces kynurenic acid. The involvement of this additional metabolic pathway during infection may be associated with impaired outcome. Because the kynurenine pathway metabolites are known to interfere with the innate and adaptive immune responses, bacterial kynurenine pathway may allow immunomodulatory interplay between bacteria and host. The existence of such interactions on the pathophysiology of *Pa* infection remains to be studied.

## Methods

### Bacterial strains

*Pa* wild type CHA strain used for experiments in this study, was isolated in 1993 from a cystic fibrosis patient four years after initial airway colonization. This strain is described in the article of Toussaint B. et al. [36] CHA has recently been included in the international reference panel of *Pa* strains. Thus, the CHA strain is maintained by the participating teams and was provided internally for the study. *Pa* wild type CHA strain and mutant strains CHAΔkynA and CHAΔkynU were grown in 5 ml of Luria Bertani broth non renewed medium (Sigma L3022, 10 g/L Tryptone, 5 g/L Yeast Extract and 5 g/L NaCl) at 37 ° C with orbital shaking (350 rpm). Inoculum standardization was performed by optical density at 600 nm (OD_600_, Ultrospec 10 Cell Density Meter, General Electrics, CT, USA) at an initial OD_600_ of 0,2.

### Mutant construction

All PCR primers used in this study have been published previously [23]. They are based on the PAO1 genome sequence (http://www.pseudomonas.com). The mutants ΔkynA and ΔkynU in this study derived from the wild-type CHA *Pa* strain. Unmarked mutants were constructed by removing an internal fragment of coding sequence by means of allelic exchange, using a Cre-lox antibiotic marker recycling method as previously described [23]. Mutant strains had no significant growth differences compared with wild-type CHA [23].

### Metabolites concentrations assessment

To assess kynurenine pathway metabolites production ex vivo, wild-type and mutants strains were grown in non-renewed LB medium. At H4, H6, H8 and H24, 500 μL of culture medium was sampled, centrifuged at 2000 g for 5 min, and immediately frozen at −80 °C for further analysis. To assess kynurenine pathway metabolites production in vivo, lungs were immediately frozen at −80 °C after extraction. Mouse lungs were homogenized in 500 μL of a half PBS - half RIPA buffer (ThermoFischer scientific) solution supplemented with protease inhibitor (Pierce™ Protease and Phosphatase Inhibitor Mini Tablets), then centrifuged (2000 g for 10 min) after resting 10 min on ice. Supernatants were immediately frozen at −80 °C for further analysis. Concentrations of tryptophan, kynurenine, kynurenic acid, 3-OH-kynurenine, anthranilate, 3-OH-anthranilate, xanthurenic acid, quinaldic acid and 8-OH-quinaldic acid were assayed, using an analytical procedure based on electrospray ionization liquid chromatography-tandem mass spectrometry (LC-ESI/MS/MS). This procedure was developed according to previously published methods, with slight modifications [37]. One hundred microliter of BAL, supernatants or culture medium were analyzed after the addition of 100 μl acetonitrile containing tryptophane-D5 at 50 000 nM, as an internal standard. The samples were mixed and centrifuged and the supernatant (100 μl) was added to deionized water (500 μl). Fifteen microliters of this mixture was injected onto an UPLC-MS/MS system (Xevo TQ-S Detector, Waters, Milford, USA) equipped with an Acquity HSS C18 column (Waters, Milford, USA). Ions of each analyzed compound were detected in a positive ion mode using multiple reaction monitoring. Chromalinks software (Waters) was used for data acquisition and processing.

### Mouse strains

Age-matched animals in the C57BL/6 J background had free access to a standard laboratory chow diet in a half-day light cycle exposure and temperature-controlled specified- pathogen free environment as determined by the Federation for Laboratory Animal Science Associations recommendations. All animal experiments were performed in an accredited establishment (B 59 – 35010) according to the governmental guidelines N886/609/CEE.

### Acute respiratory tract infection model

This model was induced by intranasal instillation of *Pa*. C57BL6/J mice were lightly anesthetized with inhaled isoflurane (Forene Abbott, Queensborough, Kent, UK), after which 50 μl of the bacterial solution was administered intranasally (5.10^6^ CFU per mouse, except for survival studies conducted with lethal inoculate: 1.10^7^ CFU per mouse). Mice were killed at 12 or 24 h. In compliance with French animal care and use in investigational research guidelines, the mice were euthanized by lethal intraperitoneal injection of 0.3 ml of 5.47 % pentobarbital (Laboratoire CEVA, Libourne, France).

### Bronchoalveolar lavage

Lungs from each experimental group were washed with a total of 1.5 ml of sterile phosphate-buffered saline (PBS). Recovered lavage fluid was pooled and centrifuged (300 g for 10 min); the cellular pellet was washed twice with PBS. Bronchoalveolar lavage (BAL) samples were filtered and immediately frozen at −80 °C after collection for metabolites concentrations assessment.

### Statistical analysis

Statistical analysis was performed using Prism 6 software (GraphPad). Results are expressed as the mean ± standard deviation (SD) or ± standard error of the mean (SEM). One-way analysis of variance followed by multiple comparison tests or unpaired *t*-test were used for all comparisons except when otherwise indicated. Significance was accepted at P less than 0.05.

## Abbreviations

BAL, bronchoalveolar lavage; KAT, kynurenine aminotransferase; KMO, kynurenine monooxygenase; KYNF, kynurenine formamidase; KYNU, kynureninase; *Pa*, *Pseudomonas aeruginosa;* PQS, pseudomonas quinolone signal; SD, standard deviation; SEM, standard error of the mean; TDO, tryptophan-2,3-dioxygenase

## Additional file

Additional file 1: Metabolites produced by the *Pa* kynurenine pathway of a clinical strain. 1. Tryptophan (A), kynurenine (B), kynurenic acid (C), anthranilate (D), and 3-0H-kynurenine (E) concentrations in growth medium supernatants of a clinical strain as determined by UPLC-MS-MS. All data from one experiment in duplicate (PPTX 164 kb)
