# Supplementary figures and images for: Tryptophan catabolism in Pseudomonas aeruginosa and potential for inter-kingdom relationship
Source: BMC Microbiol. 2016 Jul 8;16:137. doi: 10.1186/s12866-016-0756-x (PMC4938989; doi:10.1186/s12866-016-0756-x)

## Slide 1
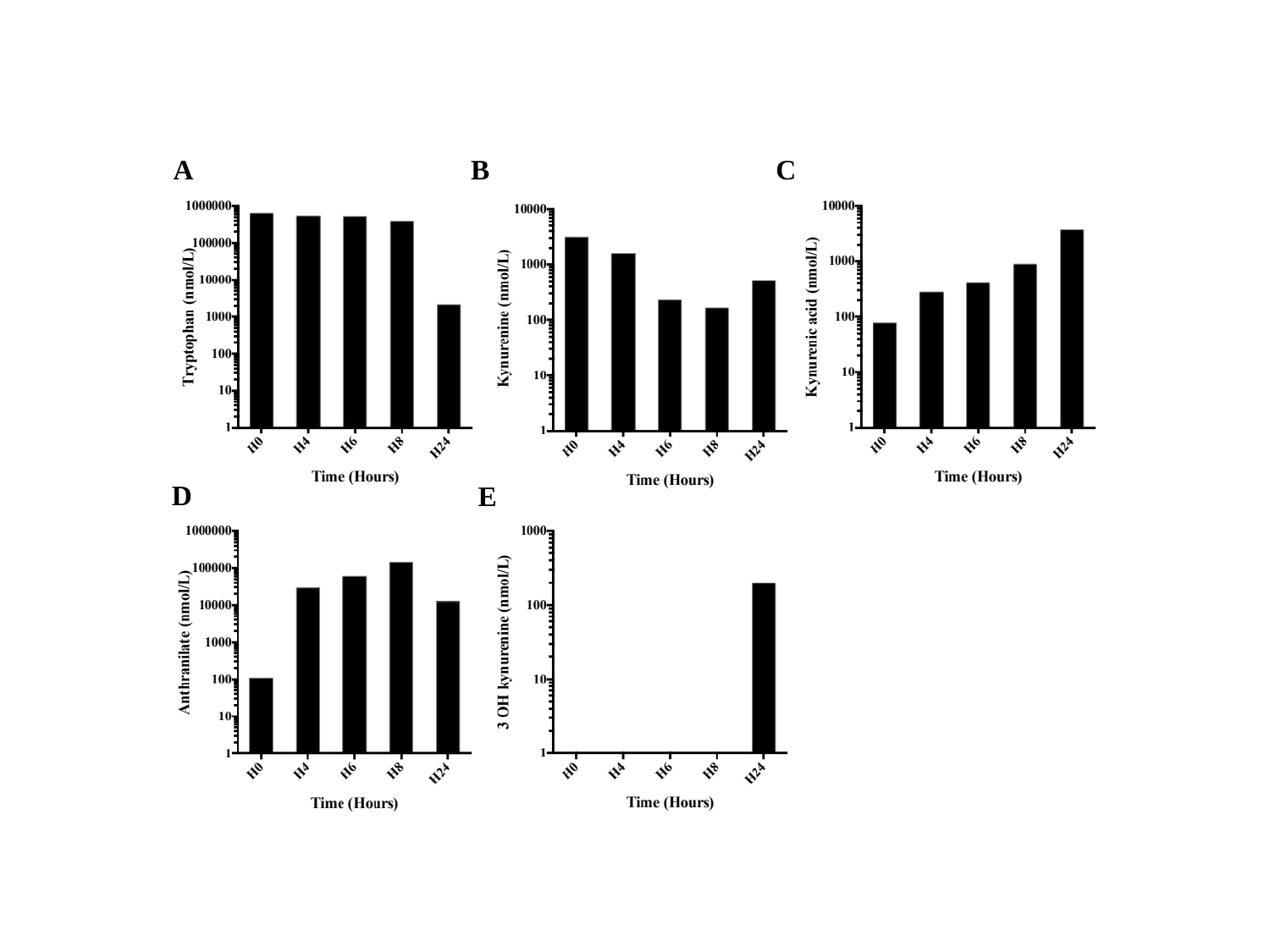

A
B
C
D
E

Supplement: Additional file 1: — Metabolites produced by the Pa kynurenine pathway of a clinical strain. 1. Tryptophan (A), kynurenine (B), kynurenic acid (C), anthranilate (D), and 3-0H-kynurenine (E) concentrations in growth medium supernatants of a clinical strain as determined by UPLC-MS-MS. All data from one experiment in duplicate (PPTX 164 kb) [file 12866_2016_756_MOESM1_ESM.pptx]
